# TFF2, a novel serum diagnostic biomarker for early pancreatic cancer

**DOI:** 10.3389/fonc.2025.1633069

**Published:** 2025-09-17

**Authors:** Xiangyu Wu, Xiuhong Huang

**Affiliations:** ^1^ Department of Orthopedic Surgery, Handan Central Hospital, Handan, China; ^2^ Department of Research, Handan Central Hospital, Handan, China

**Keywords:** TFF2, CA199, serum biomarker, noninvasive, pancreatic cancer

## Abstract

**Background:**

Early detection and intervention are critical for improving the prognosis of pancreatic cancer (PC), but effective screening tests remain unavailable.

**Methods:**

A retrospective analysis was conducted on 92 PC cases, 50 benign pancreatic disease cases, 92 periampullary adenocarcinoma cases, and 92 healthy controls from September 2020 to September 2024 at Handan Central Hospital. Serum levels of CA199 and CEA were measured, and their diagnostic performance was evaluated by the area under the receiver operating characteristic (ROC) curve (AUC). Additionally, publicly available cancer genome datasets were analyzed to identify specific serum biomarkers for early PC, and clinical serum samples were collected to validate their expression and diagnostic utility.

**Results:**

CA199 and CEA effectively differentiated PC from benign pancreatic diseases and normal controls. However, they demonstrated limited value for distinguishing PC and periampullary carcinoma, with AUC values of 0.571 and 0.604, respectively. Trefoil factor 2 (TFF2), a gene encoding exocrine protein, was found to be specifically upregulated in pancreatic intraepithelial neoplasia and PC, with no significant expression observed in benign pancreatic diseases, cholangiocarcinoma, or hepatocellular carcinoma. Importantly, serum TFF2 levels were significantly elevated in the PC group, with AUC values of 0.947 for distinguishing PC from normal controls and 0.856 for differentiating it from periampullary adenocarcinoma, outperforming CA199 and CEA. The combination of TFF2 enhanced accuracy of CA199 and CEA to discriminate PC from periampullary adenocarcinoma.

**Conclusions:**

Serum TFF2 is a promising test for early screening of PC and may enhance diagnostic performance when combined with CA199 and CEA.

## Introduction

Pancreatic cancer (PC) is a leading cause of cancer death worldwide, marked by an insidious onset, aggressive progression, and resistance to therapeutic interventions (1). The prognosis for PC remains dismal, with a five-year overall survival rate of approximately 8%. This is primarily due to its deep anatomical location and the lack of early, recognizable symptoms, which often leads to delayed diagnosis and limited therapeutic interventions. If detected early, while the tumor is still confined to the pancreas, surgical resection offers the potential for cure. And the five-year survival rate for patients with tumors confined to the duct epithelium can approach 100% when the tumors are < 1cm (2). However, only 10% of PC cases are diagnosed at an early stage (3, 4). Thus, early detection is crucial for improving the prognosis of patients with PC.

The early diagnosis of PC remains a formidable challenge due to its asymptomatic progression and deep anatomical location. Current diagnostic approaches for PC commonly rely on a combination of imaging modalities, such as computed tomography (CT), magnetic resonance imaging (MRI), and endoscopic ultrasound (EUS), serum biomarkers and biopsy-based histopathology (5–7). Imaging techniques provide detailed structural visualization, but their ability to detect small tumors is limited, particularly in visualizing pancreatic intraepithelial neoplasia (PanIN) prior to the development of invasive cancer. Molecular evidence indicates most pancreatic cancers arise from PanIN, which unfortunately are not identifiable by current imaging modalities (8, 9). Furthermore, although biopsy-based histopathology, such as endoscopic ultrasound-guided fine-needle aspiration (EUS-FNA), remains the gold standard for definitive diagnosis, it is time consuming, invasive and carries associated risks, including iatrogenic pancreatitis, hemorrhage, infection, perforation, and needle tract seeding (10–12). Additionally, their accessibility is limited by high costs, the requirement for specialized equipment, and trained personnel. Therefore, it is urgent to find a low-cost, high-accuracy, and noninvasive screening tool capable of detecting premalignant conditions with a high risk of PC, while also minimizing unnecessary diagnostic procedures.

Although pancreatic cancer-specific blood-based biomarkers involving circulating tumor cells (CTCs), circulating tumor DNA (ctDNA), microRNAs, exosomes, and serum proteins are drawing more and more attention, none of these approaches have yet been adopted into clinical practice (13–16). Currently, carbohydrate antigen 199 (CA199) is the routinely used serum biomarker for PC management and is the only serum biomarker approved by the U.S. Food and Drug Administration (FDA) for PC. In addition, carcinoembryonic antigen (CEA) and carbohydrate antigen 125 (CA125) are often used in combination to diagnose pancreatic cancer (17, 18). CA199 is widely used as a key prognostic marker for assessing therapeutic efficacy, monitoring metastasis, and predicting survival in patients with advanced PC (19–21). However, its application in early diagnosis remains limited by insufficient sensitivity and specificity. Additionally, while CA199 exhibits high sensitivity for diagnosing advanced PC, it has no advantages regarding specificity, particularly in cases of biliary obstruction or inflammation. Hence, there is an urgent need to identify a highly sensitive and specific biomarker complementary to CA199 for enhancing the early PC screening.

In the current study, we aimed to identify a novel serum biomarker with improved discriminative accuracy for early PC detection. We began by assessing the expression levels of CA199 and CEA across various diseases and evaluating their discriminative performance for PC. Next, we identified an early-stage PC-specific serum biomarker through bioinformatics analysis of available cancer genome datasets, followed by validation of its expression in clinical samples using enzyme-linked immunosorbent (ELISA) assay. Finally, we evaluated the discriminative performance of individual marker in combination with CA199 and CEA in patients with PC and periampullary adenocarcinoma.

## Materials and methods

### Patients and samples

Patients with pancreatic cancer and periampullary adenocarcinoma, including extrahepatic biliary, duodenal, and ampullary adenocarcinoma, who were hospitalized at Handan Central Hospital from September 2020 to September 2024 were included in the study. Additionally, there were 19 patients with chronic pancreatitis and 31 with benign pancreatic masses involved as the benign controls, and 92 healthy individuals from the physical examination center as the normal controls. Among the 31 patients with benign pancreatic masses, 13 had cystadenomas, 2 had intraductal papillary mucinous neoplasms, and 16 had solid-pseudopapillary neoplasms (SPN). Diagnosis of pancreatic masses was confirmed by surgical resection, while chronic pancreatitis was diagnosed based on serological combined with imaging examinations. None of the participants had received prior cancer therapy or had concurrent primary malignancies at other sites, whose clinical data was fully available. The groups were matched by age and gender. Serum levels of CA199 and CEA were measured before any treatment. Meanwhile, serum samples from patients with PC, CCA, duodenal adenocarcinoma, and ampullary adenocarcinoma were obtained from the clinical laboratory for ELISA analysis. All serum samples were collected prior to any treatment. The study was censored and approved by the Ethics Committee of Handan Central Hospital.

### Transcriptome data acquisition and processing

The GSE43288 dataset, comprising normal, PanIN, and PC samples, was obtained from the Gene Expression Omnibus (GEO) database (https://www.ncbi.nlm.nih.gov/geo/). Additionally, the RNA-seq data of TCGA_PAAD dataset were retrieved from the Cancer Genome Atlas (TCGA) database (https://portal.gdc.cancer.gov/). These datasets were selected for the purpose of screening potential serum biomarkers. All transcriptome data were normalized, standardized, and log2-transformed using the “limma” R package, which fits linear models to expression values and employs empirical Bayes moderation to improve variance estimates, particularly in studies with small sample sizes.

### Identification of serum markers for pancreatic cancer

To identify potential serum biomarkers for early PC, differential gene analysis on GSE43288 and TCGA_PAAD cohort was initially conducted utilizing the “limma” package of R software with threshold of |log2 fold change (logFC)|≥1 and a false discovery rate (FDR)<0.05. Differentially expressed genes (DEGs) were defined as up- or down-regulated when logFC ≥1 or ≤-1, respectively, with FDR*<*0.05. Subsequently, the genes encoding exocrine protein were obtained from the Human Protein Atlas (HPA: https://www.proteinatlas.org/) database and intersected with the upregulated DEGs to further determine potential serum biomarkers. Additionally, TIMR2.0 (http://timer.comp-genomics.org/) was also used to analyze the mRNA expression levels of markers in different cancers across TCGA. All abbreviations were showed in **Supplementary Table 1**. Finally, the diagnostic efficiency of candidate marker and tumor markers for PanIN and PC was assessed with receiver operating characteristic (ROC) curves using the “pROC” package.

### Single-cell analysis

Because bulk transcriptomic data reflect only overall tissue expression, we performed single-cell analysis to identify the cellular origin of the marker gene, providing evidence for its specificity and potential as a serum biomarker. The scRNA-seq data GSE155698 was downloaded from the GEO database, and 16 PC tissue samples, 3 adjacent normal pancreas samples were selected to construct “Seurat” objects using the “Seurat” R package (v4.3.0). Initially, data were filtered to only retain cells with at least 200 genes and genes that expressed in more than 3 cells. Low-quality cells with <200 or >7000 transcripts and >10% mitochondrial genes were then manually filtered. And 41,010 cells were retained. Data were normalized and scaled, and variable genes were identified via the FindVariableFeatures function. Subsequently, principal component analysis (PCA) was performed for dimension reduction, and the number of selected dimensions was set as 15. Then cell clusters were identified by the t-distribution stochastic neighbor embedding (t-SNE) algorithms, and were defined with several well-known markers. Finally, the distribution and expression of marker gene, TFF2, was visualized.

### Expression analysis of TFF2 by multiple datasets

To further evaluate and validate the expression levels of marker genes in diverse cancers, gene expression microarray datasets, including GSE15471, GSE16515, GSE32676, GSE101462, GSE43795, GSE143754, GSE76297, GSE26566, GSE89377, and GSE39409 were obtained from the GEO database. Probes were mapped to their corresponding gene symbols using platform-specific annotation files. Datasets annotated with the same platform were subsequently integrated into new cohorts. Batch effects across the datasets were corrected using the removeBatchEffect function from the “limma” package in R. Additionally, the performance of marker genes in distinguishing PC and controls was assessed using ROC curves. Detailed sample information of the datasets mentioned above was shown in **Supplementary Table 2**.

### Enzyme-linked immunosorbent assays

An ELISA kit for human TFF2 (Cusabio) was utilized to quantify serum TFF2 levels in patients with PC, CCA, duodenal adenocarcinoma, ampullary adenocarcinoma, and healthy individuals following the manufacturer’s protocol. The optical density was measured at 450 nm with a 570 nm wavelength correction.

### Statistical analysis

All statistical analyses and graphical visualizations were performed with R software 4.1.2 or GraphPad Prism 8.0. The R packages and statistical methods used in this study were described in detail throughout the manuscript. Quantitative data were shown as the mean ± standard error of the mean (SEM) or medians (interquartile range, IQR). Student’s t test or Wilcoxon rank sum test was used to calculate the statistical significance of normal and skewed distribution variables between two groups, respectively. One-way analysis of variance (ANOVA) test or Kruskal-Wallis test was performed to analyze the significance of continuous variables among groups ≥3. Categorical variables were described as count (percentage) and compared using the chi-square test. Discrimination was evaluated by plotting ROC curves and calculating the area under curve (AUC). The optimal cut-off value was determined using the Youden index. *P<*0.05 was considered statistically significant.

## Results

### Expression levels of serum CA199 and CEA

**Figure 1:**
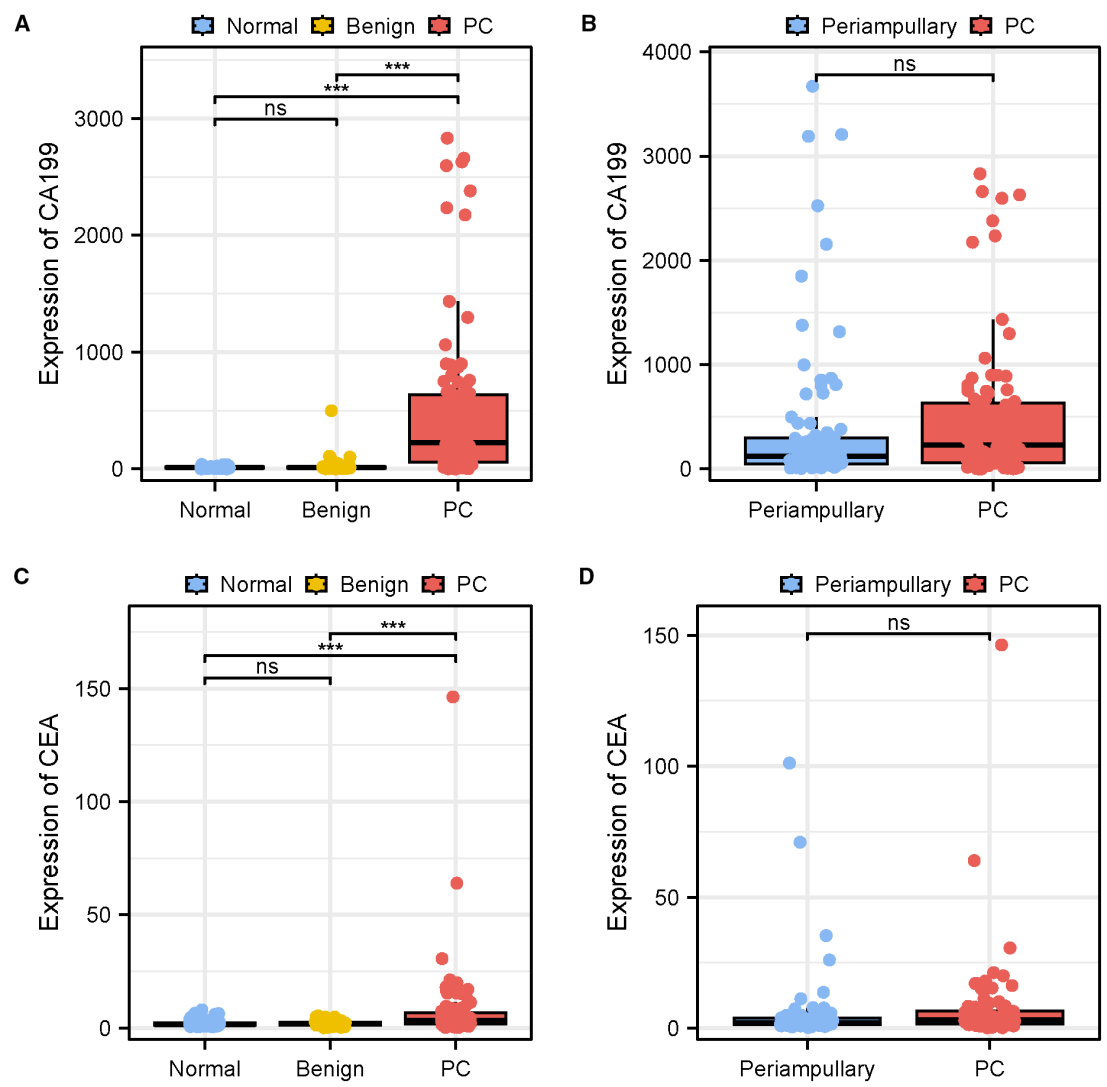
Comparison of CA199 and CEA serum levels between PC group and controls. **(A, C)** Serum CA199 and CEA expression levels from patients with PC and benign pancreatic diseases, and healthy individuals. **(B, D)** Serum CA199 and CEA expression levels from patients with PC and periampullary adenocarcinoma. Data are shown as the medians (IQR). Significance is indicated as follows: *P<0.05, **P<0.01, ***P<0.001; ns represents no significant difference. Significance is indicated as follows: ***P<0.001; ns represents no significant difference.

A total of 92 patients with PC and 92 patients with periampullary adenocarcinoma, including 33 with CCA, 31 with ampullary carcinoma, and 28 with duodenal adenocarcinoma were enrolled in this study. Besides, there were 50 patients with benign pancreatic diseases and 92 healthy individuals included as benign and healthy control groups, respectively. Baseline characteristics of the study population were presented in **Table 1**. The distributions of CA199 and CEA in different groups were first explored. The median of CA199 was 10.72 IU/mL (6.778-15.37) for normal controls, 12.07 IU/mL (8.393-18.85) for benign controls, 121.7 IU/mL (49.53-297.5) for tumor controls, and 227.3 IU/mL (57.53-635.2) for pancreatic cancer patients. Statistical analysis revealed that the expression levels of CA199 were significantly higher in the PC group, compared to normal and benign control groups (*P<*0.05, P<0.001 **Figure 1A**). In contrast, within the control groups, CA199 expression was not significantly different among patients with chronic pancreatitis, those with benign pancreatic masses, and normal controls (**Figure 1A**). Similarly, no significant difference in CA199 expression was observed between PC patients and the tumor control group composed of periampullary carcinomas (**Figure 1B**). As for CEA, the median of CEA was 1.535 ng/mL (1.098-2.168) for normal controls, 1.705 ng/mL (1.230-2.508) for benign controls, 2.175 ng/mL (1.385-3.948) for tumor controls, and 3.205 ng/mL (1.558-7.150) for pancreatic cancer patients. As illustrated in **Figure 1C**, CEA expression between the PC and control groups was significantly different (*P<*0.05, P<0.001). However, there was no significant difference in CEA expression levels between pancreatic cancer and periampullary adenocarcinoma patients (**Figure 1D**).

**Table 1:** Baseline characteristics of the included patients.

Characteristic	PC (N=92)	Benign disease (N=50)	Periampullary adenocarcinoma (N=92)	Healthy (N=92)
Age, mean	63.16	59.52	64	60.13
Gender				
Male	49 (53.26%)	25 (50.00%)	54 (58.70%)	36 (42.35%)
Female	43 (46.74%)	25 (50.00%)	38 (41.30%)	56 (56.57%)
Tumor stage				
Stage I-II	92 (100%)	0	92 (100%)	0
Stage III-IV	0	0	0	0
CA199, IU/mL	227.3 (57.53-635.2)	12.07 (8.393-18.85)	121.7 (49.53-297.5)	10.72 (6.778-15.37)
CEA, ng/mL	3.205 (1.558-7.150)	1.705 (1.230-2.508)	2.175 (1.385-3.948)	1.535 (1.098-2.168)

Age and gender were matched between study groups. PC, pancreatic cancer; CA199, carbohydrate antigen 199; CEA, carcinoembryonic antigen.

### Comparison of the diagnostic performance among CA199 and CEA

**Figure 2:**
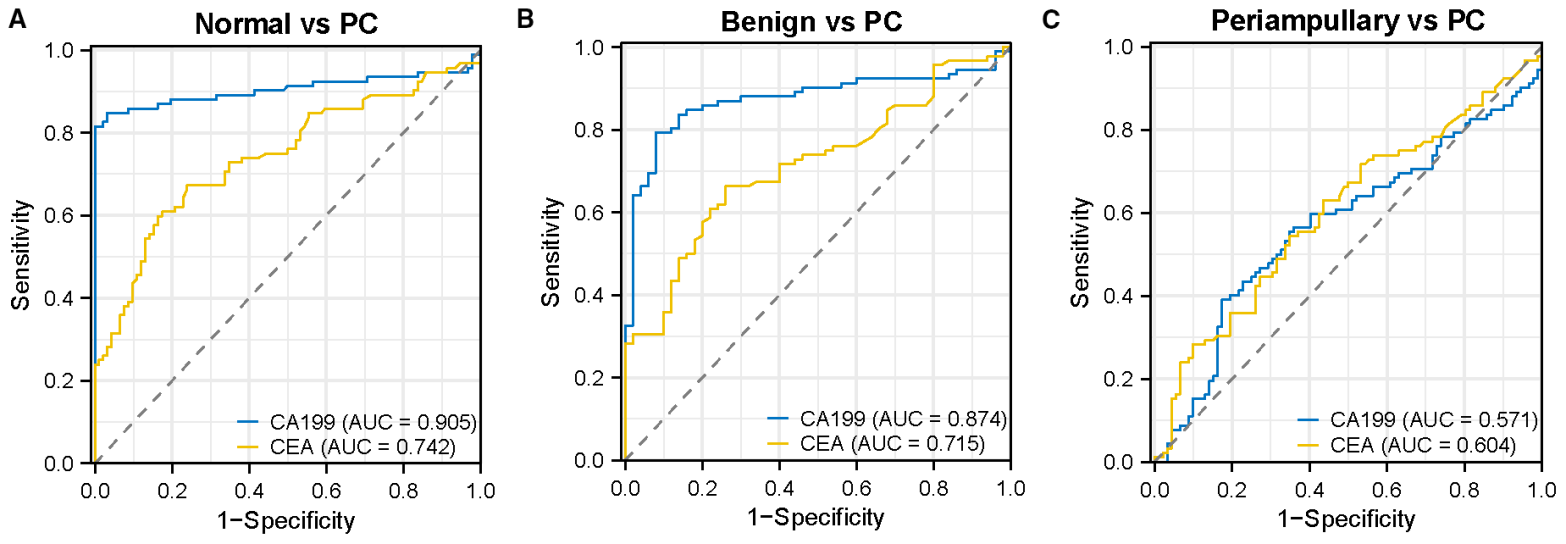
Evaluation of the diagnostic performance of serum CA199 and CEA. **(A–C)** ROC curves illustrating the efficacy of serum CA199 and CEA in discriminating PC from normal controls, benign controls, and periampullary adenocarcinoma.

To evaluate the diagnostic performance of CA199 and CEA for distinguishing PC cases from controls, ROC curves were constructed. CA199 demonstrated a superior ability to differentiate PC from normal and benign controls, with AUC values of 0.905 (95% CI 0.851-0.958, *P<*0.0001, **Table 2**, **Figure 2A**) and 0.874 (95% CI 0.813-0.934, *P<*0.0001, **Table 2**, **Figure 2B**), respectively. These values significantly exceeded those of CEA, which yielded AUC values of 0.742 (95% CI 0.670-0.815, *P<*0.0001, **Table 2**, **Figure 2A**) and 0.715 (95% CI 0.631-0.799, *P<*0.0001, **Table 2**, **Figure 2B**). Using the Youden index, the optimal cut-off values for CA199 were determined as >29.54 IU/mL and >46.69 IU/mL, with corresponding sensitivities of 84.78% and 79.35% and specificities of 96.74% and 92%, respectively (**Table 2**). In comparison, the optimal cut-off values for CEA were >2.185 ng/mL and > 2.260 ng/mL, resulting in sensitivities of 67.39% and 66.3% and specificities of 76.09% and 74%, respectively (**Table 2**). We next assessed the ability of CA199 and CEA to differentiate PC from periampullary adenocarcinoma. Both biomarkers exhibited limited discriminative performance, with AUC values of 0.571 (95% CI 0.486-0.655, **Table 2**, **Figure 2C**) and 0.604 (95% CI 0.522-0.686, **Table 2**, **Figure 2C**), respectively. Using optimal thresholds of CA199 >444.9 IU/mL and CEA >2.995 ng/mL, the sensitivity and specificity for CA199 were 39.13% and 82.61%, while those for CEA were 54.35% and 65.22%, respectively (**Table 2**). Our findings confirmed the robust diagnostic potential of CA199 in PC detection, but also revealed its limitations in distinguishing PC from periampullary adenocarcinoma. As a result, there was an urgent need to identify and explore more reliable and specific biomarkers that could effectively differentiate PC from other malignancies.

**Table 2:** Diagnostic performance of CA199 and CEA.

Biomarker	Group	Cut-off	Sens%	Spec%	PPV%	NPV%	AUC (95%CI)	*P* value
CA199	Normal VS PC	29.54	84.78	96.74	96.30	86.41	0.905 (0.851- 0.958)	<0.0001
	Benign vs PC	46.69	79.35	92.00	94.81	70.77	0.874 (0.813- 0.934)	<0.0001
	Periampullary vs PC	444.9	39.13	82.61	69.23	57.58	0.571 (0.486-0.655)	0.097
CEA	Normal VS PC	2.185	67.39	76.09	73.81	70.00	0.742 (0.670- 0.815)	<0.0001
	Benign vs PC	2.260	66.30	74.00	82.43	54.41	0.715 (0.631-0.799)	<0.0001
	Periampullary vs PC	2.995	54.35	65.22	60.98	58.82	0.604 (0.522-0.686)	<0.05

All cut-offs maximize the Youden index. Sens, sensitivity; Spec, specificity; PPV, positive predictive value; NPV, negative predictive value; AUC, the area under the curve.

### Identification of serum markers for pancreatic cancer

**Figure 3:**
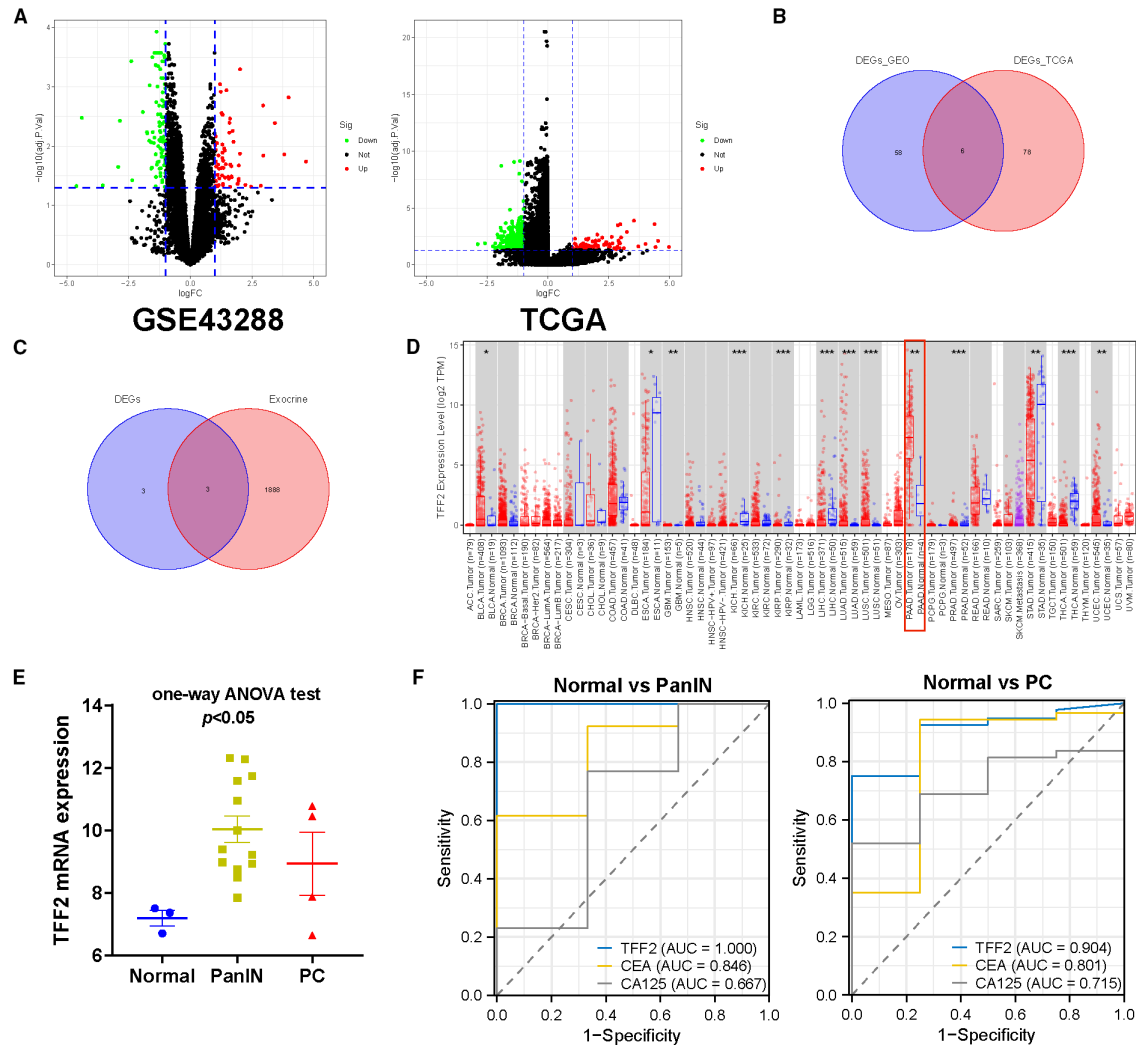
Identification of serum markers for early pancreatic cancer. **(A)** The volcano plots of DEGs. **(B)** Venn diagram showing 6 intersection genes from upregulated DEGs in GSE43288 and the TCGA_PAAD cohort. **(C)** Venn diagram showing 3 intersection genes from the DEGs and genes encoding exocrine proteins of the HPA database. **(D)** Gene expression levels of TFF2 in different cancers across TCGA. **(E)** Gene expression levels of TFF2 in different pathological stages from GSE43288. **(F)** ROC analysis illustrating the diagnostic performance of TFF2 and tumor markers for PanIN and PC.

To identify potential serum biomarkers for early pancreatic cancer, we initially conducted a differential gene expression analysis. By comparing PanIN with normal tissues, 155 DEGs were identified in the GSE43288 dataset, including 64 upregulated genes and 91 downregulated genes. Similarly, 347 DEGs, including 84 upregulated genes and 263 downregulated genes, were identified between PC and normal tissues in the TCGA_PAAD cohort. The differential gene expression profiles were visualized by volcano plots (**Figure 3A**). Subsequently, 6 intersection genes, including TFF1, AGR2, S100P, TFF2, MUC1, FXYD3 were identified between upregulated DEGs from GSE43288 and those from the TCGA_PAAD cohort using a Venn diagram (**Figure 3B**). To find biomarkers used as noninvasive tests, we intersected 6 genes with the gene set encoding exocrine proteins from the HPA database. Eventually, 3 genes, including TFF1, TFF2, and MUC1 were retained (**Figure 3C**). To further identify biomarkers specifically elevated in pancreatic cancer, we utilized TIMR2.0 to analyze and compare the expression levels of intersection genes in different cancers across TCGA. Notably, TFF2 was significantly upregulated only in pancreatic cancer, whereas it was either downregulated or exhibited no significant changes in other gastrointestinal cancers (**Figure 3D**). In contrast, the remaining two intersection genes exhibited upregulation across multiple cancer types, particularly in CCA and HCC (**Supplementary Figure S1**). Accordingly, TFF2 was selected for subsequent analysis due to its cancer specificity. Meanwhile, analysis of the GSE43288 dataset revealed TFF2 expression was significantly different among pathological stages (**Figure 3E**). These results indicated that TFF2 expression was altered during early pancreatic carcinogenesis, highlighting its potential utility as a biomarker for early detection. Finally, we assessed the diagnostic performance of TFF2 alongside conventional tumor markers, including CEA (CEACAM5) and CA125 (MUC16), for PanIN and PC through ROC curve analysis, and the AUC values were 1.000 and 0.904, significantly outperforming other tumor markers (**Figure 3F**). In summary, TFF2 was identified as a biomarker encoding an extracellular protein with robust diagnostic efficacy, underscoring its potential as a noninvasive indicator for early detection.

### Expression validation of TFF2 by single-cell analysis

**Figure 4:**
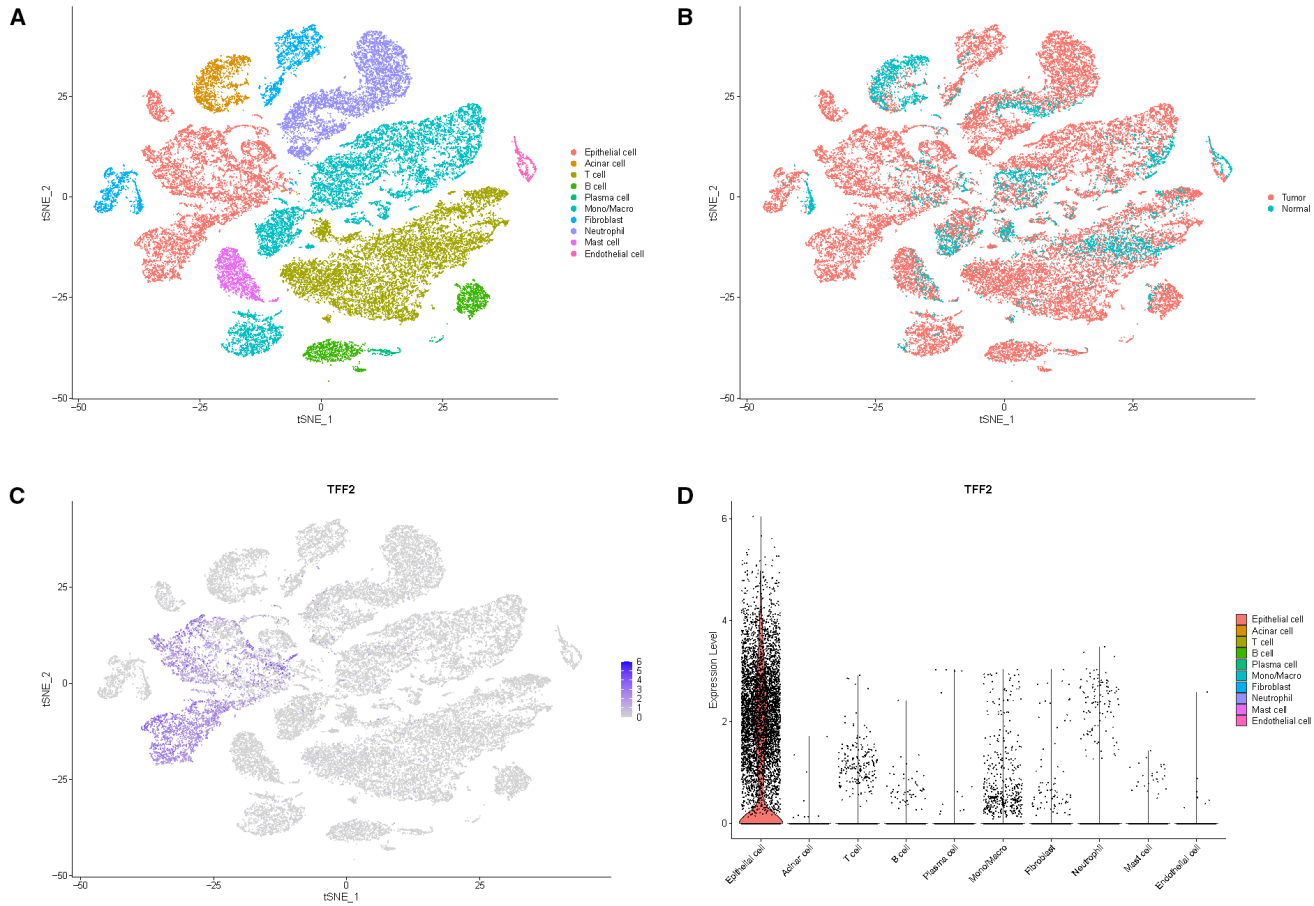
Validation of TFF2 expression pattern by single-cell analysis. **(A, B)** Cluster diagrams based on cell types and sample sources. **(C)** Feature plot of TFF2 expression by single-cell analysis. **(D)** Violin plot illustrating the distribution of TFF2 expression at the single-cell level.

To determine the cell types responsible for TFF2 secretion, we analyzed the pancreatic cancer single-cell RNA sequencing dataset GSE155698. After quality control, 41,010 cells were retained for analysis. The t-SNE algorithms revealed 40 clusters, corresponding to 10 cell types: epithelial cell, acinar cell, T cell, B cell, plasma cell, monocyte/macrophage, fibroblast, neutrophil, mast cell, and endothelial cell (**Figure 4A**). The distribution of TFF2 expression according to the sample sources and cell types was shown in **Figures 4B–D**. These results indicated that TFF2 was predominantly secreted by epithelial cells and exhibited high expression levels in tumor samples. Given that PC arises from the malignant transformation of pancreatic ductal epithelial cells, single-cell analysis results further support the potential of TFF2 as a reliable serum biomarker for PC.

### Validation of the diagnostic efficacy of TFF2 by multiple datasets

**Figure 5:**
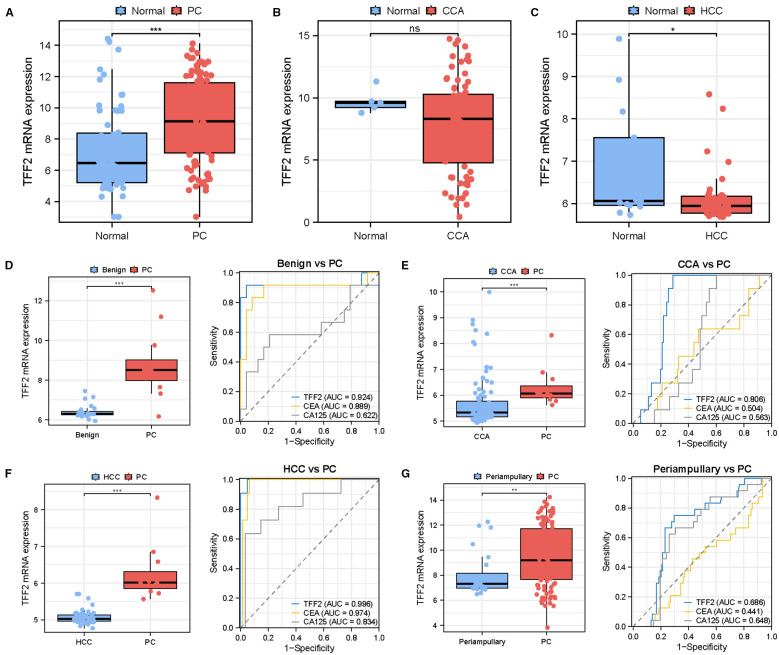
Validation of the diagnostic efficacy of TFF2. **(A–C)** Comparison of TFF2 mRNA expression across multiple datasets: in normal and PC tissues (GSE15471, GSE16515, and GSE32676), CCA tissues (GSE26566), and HCC tissues (GSE89377). **(D–G)** Statistical analyses and ROC curve assessments of TFF2 and tumor markers at the mRNA level, comparing PC with benign controls, CCA, HCC, and periampullary adenocarcinoma. Data are shown as the mean ± SEM. Significance is indicated as follows: *P<0.05, **P<0.01, ***P<0.001; ns represents no significant difference.

To evaluate the specificity of TFF2 as a diagnostic marker for pancreatic cancer, we examined its expression levels across publicly available datasets from the GEO database. Analyzing a cohort of 100 tumor samples and 62 normal samples, including datasets GSE15471, GSE16515, and GSE32676, a significant upregulation of TFF2 was observed in PC samples compared to normal control (*P<*0.05, P<0.001, **Figure 5A**). The expression of TFF2 in CCA and HCC was validated in the GSE26566 and GSE89377 datasets, respectively, which was consistent with the findings from the TCGA database (**Figures 5B, C**). Meanwhile, chronic pancreatitis (GSE101462) and SPN (GSE43795) were also included and merged as a benign control in the study. The results showed that TFF2 expression was significantly elevated in the PC group compared to the benign control, and TFF2 demonstrated the best performance in distinguishing pancreatic benign conditions from malignancies, with an AUC of 0.924 (**Figure 5D**). We next assessed the accuracy of TFF2 in distinguishing PC from CCA and HCC using a cohort derived from the merged GSE143754 and GSE76297 datasets. TFF2 mRNA expression was significantly upregulated in PC patients compared to those with CCA or HCC (P<0.001, **Figures 5E, F**). ROC curve analysis revealed that TFF2 achieved AUC values of 0.806 and 0.996, respectively, for differentiating PC from CCA and HCC, significantly outperforming CEA and CA125 (**Figures 5E, F**). Additionally, the GSE15471, GSE16515, GSE32676, and GSE39409 datasets were integrated to further investigate the diagnostic potential of TFF2 in differentiating PC from periampullary adenocarcinoma. Statistical analysis revealed that TFF2 expression was upregulated in PC, yielding an AUC of 0.686, which demonstrated superior diagnostic performance relative to CEA and CA125 (**Figure 5G**). As a result, TFF2 effectively discriminated between PC and other controls, highlighting its potential as a promising diagnostic marker for PC.

### Validation of diagnostic efficacy of TFF2 in serum samples

**Figure 6:**
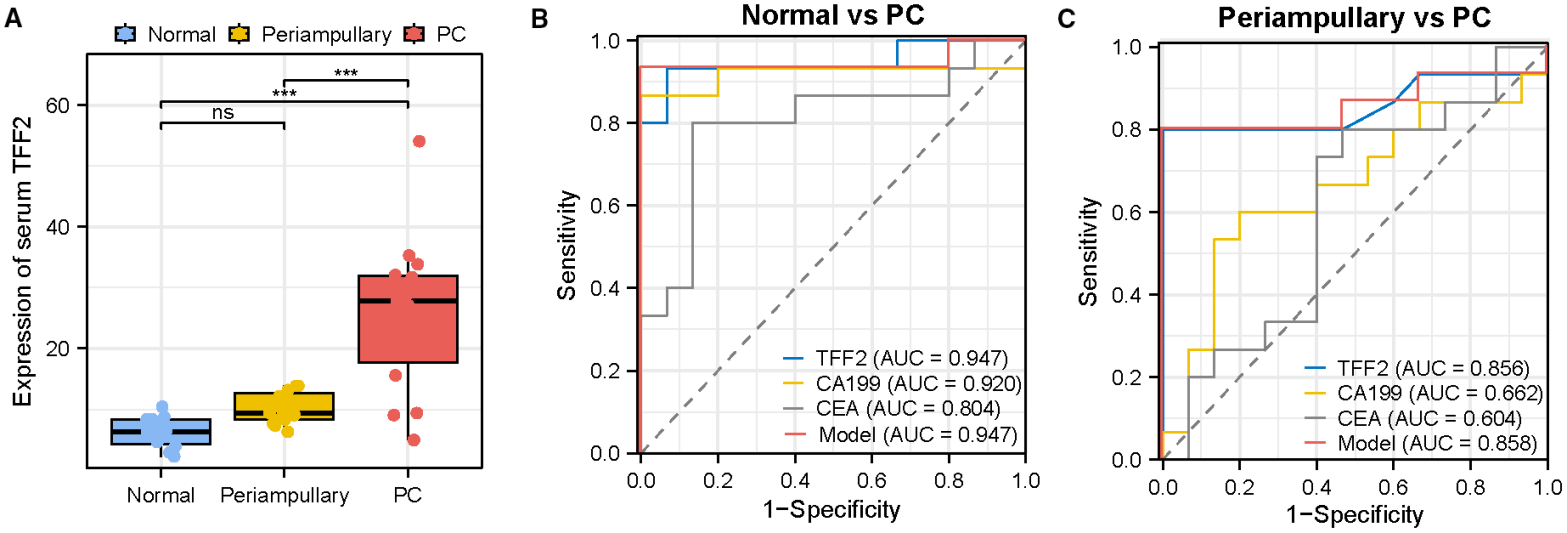
Validation of Diagnostic efficacy of serum TFF2. **(A)** Serum TFF2 expression levels from normal controls, periampullary adenocarcinoma patients, and PC patients. Data are shown as the mean ± SEM. Significance is indicated as follows: *P<0.05, **P<0.01, ***P<0.001; ns represents no significant difference. Significance is indicated as follows: ***P<0.001; ns represents no significant difference. **(B, C)** ROC analysis results for serum TFF2, CA199, CEA and the combined biomarker panel in discriminating PC patients from healthy individuals and periampullary adenocarcinoma patients, respectively.

To evaluate whether TFF2 could be used as a serum marker for PC, the levels of TFF2 in serum samples from 15 patients with PC, 15 patients with periampullary adenocarcinoma, including 6 CCA, 6 ampullary carcinoma, and 3 duodenal adenocarcinoma patients, and 15 healthy controls were determined by a quantitative ELISA assay. No statistically significant difference in serum TFF2 levels was observed between the normal and periampullary adenocarcinoma groups. However, serum TFF2 concentrations were elevated in PC patients compared to both normal individuals and periampullary adenocarcinoma patients (*P<*0.001, **Figure 6A**). The corresponding ROC curves were performed to further illustrate the diagnostic efficacy of serum TFF2, yielding an AUC of 0.947 for differentiating PC from normal individuals and 0.856 for distinguishing PC from periampullary adenocarcinoma. These values notably surpassed those of CA199 and CEA (**Figures 6B, C**, **Table 3**). Moreover, we also constructed ROC curves for the combination of biomarkers. This combined biomarker panel significantly distinguished PC from normal controls and periampullary adenocarcinoma, with AUC values of 0.947 (**Figure 6B**, **Table 3**) and 0.858 (**Figure 6C**, **Table 3**), respectively, outperforming CA199 and CEA when used individually. As a result, serum TFF2 effectively distinguished PC from other controls, which was complementary to CA199 and CEA for enhancing the PC diagnosis.

**Table 3:** Diagnostic performance of serum markers.

Biomarker	Group	Cut-off	Youden index	Sens%	Spec%	PPV%	NPV%	AUC (95%CI)
TFF2	Normal VS PC	8.875	0.8666	93.33	93.33	93.33	93.33	0.947 (0.856-1.000)
	Periampullary vs PC	14.72	0.8000	80.00	100.0	100.0	83.33	0.856 (0.694-1.000)
CA199	Normal VS PC	38.32	0.8667	86.67	100.0	100.0	88.24	0.920 (0.788-1.000)
	Periampullary vs PC	297.2	0.4000	60.00	80.00	75.00	66.67	0.662 (0.455-0.869)
CEA	Normal VS PC	3.040	0.6667	80.00	86.67	85.71	81.25	0.804 (0.634-0.975)
	Periampullary vs PC	3.040	0.3333	80.00	53.33	63.16	72.73	0.604 (0.391-0.818)
Model	Normal VS PC	0.085	0.9333	93.33	100.0	100.0	93.75	0.947 (0.841-1.000)
	Periampullary vs PC	0.146	0.8000	80.00	100.0	100.0	83.33	0.858 (0.697-1.000)

All cut-offs maximize the Youden index. Sens, sensitivity; Spec, specificity; PPV, positive predictive value; NPV, negative predictive value; AUC, the area under the curve.

## Discussion

Pancreatic cancer remains among the most aggressive and lethal malignancies, with alarmingly low survival rates, which can be improved through early and accurate diagnosis. Currently, CA199 and CEA are the routinely used serum biomarker for PC management (22). In the study, we implemented a case-control design to investigate serum levels of CA199 and CEA, aiming to determine their diagnostic performance in distinguishing PC from controls. Our findings confirmed the robust diagnostic potential of CA199 in PC detection, but also revealed its limitations in distinguishing PC from periampullary adenocarcinoma. Similarly, although CEA was explored as an alternative, its diagnostic performance remains consistently inferior to CA199, with notably lower sensitivity and specificity.

Differentiating PC from periampullary adenocarcinoma, including CCA, duodenal adenocarcinoma, and ampullary carcinoma, is critical in clinical practice due to their distinct treatment strategies and prognoses. Although these malignancies share overlapping clinical presentations and imaging characteristics, they originate from anatomically proximate but biologically distinct regions, which makes precise diagnosis a cornerstone for tailoring effective treatment strategies and optimizing patient outcomes. Current diagnostic approaches, including EUS, magnetic resonance cholangiopancreatography (MRCP), endoscopic retrograde cholangiopancreatography (ERCP), and biopsy-based histopathology play central roles. However, these methods are frequently limited by sampling errors, associated risks, overlapping histological characteristics, and the inherent resolution constraints of non-invasive imaging techniques (23, 24). Serum biomarkers, including CA199 and CEA, are routinely utilized but exhibit suboptimal specificity and sensitivity in cases of biliary obstruction or inflammation (25–27). As a result, there is an urgent need to identify a novel biomarker with improved discriminative accuracy to compensate for the shortcomings of CA199 and CEA.

In this study, we conducted transcriptomic analysis to identify DEGs between PC and normal tissues. Upregulated DEGs were intersected with genes encoding extracellular proteins obtained from the HPA database to further determine candidate marker genes. A subsequent pan-cancer analysis validated TFF2 as a highly specific marker for PC. Furthermore, we evaluated the diagnostic potential of TFF2 in comparison with conventional tumor markers at the mRNA level through ROC curve analysis. TFF2 achieved an AUC of 0.904 in diagnosing PC, significantly outperforming other tumor markers. Meanwhile, we performed single-cell analysis to determine TFF2 expression pattern. The results revealed that TFF2 was predominantly secreted by epithelial cells and exhibited elevated expression in tumor samples, highlighting its potential of TFF2 as a blood biomarker for PC. Next, we validated its expression levels across multiple datasets from the GEO database. TFF2 was found to be significantly overexpressed in pancreatic cancer but not in CCA or HCC, suggesting its higher specificity for PC. This distinct expression pattern further strengthens its feasibility as a robust biomarker to discriminate pancreatic cancer from other malignancies. Also, TFF2 expression levels in PC were notably higher than those observed in benign pancreatic conditions, CCA, HCC, and periampullary adenocarcinoma, with statistically significant differences. Importantly, ROC analysis further confirmed that TFF2 achieved superior diagnostic accuracy for PC, surpassing conventional tumor markers with higher AUC values.

Preclinical analyses of differentially expressed genes from the TCGA_PAAD cohort and GEO datasets have suggest that TFF2 are significantly upregulated in PC and may be a possible serum marker. Therefore, we next conducted experiments using clinical samples to confirm the discriminative power of serum TFF2. We measured TFF2 levels in serum samples from healthy controls, as well as patients with PC and periampullary adenocarcinoma and found that TFF2 was specifically overexpressed in PC patients, but not in periampullary adenocarcinoma patients or healthy controls. Notably, ROC analysis demonstrated superior diagnostic performance of serum TFF2 for PC, with AUC values exceeding those of CA199 and CEA. In particular, the AUC for distinguishing PC from periampullary adenocarcinoma reached an impressive 0.856. When combined with CA 199 and CEA, the AUC further increased to 0.858. Taken together, our findings indicate that serum TFF2 has the potential to enhance the diagnostic accuracy for PC and may serve as a promising non-invasive test.

Pancreatic cancer ranks the deadliest cancer with a dismal prognosis, which is primarily attributed to its hidden anatomical location and the absence of early, recognizable symptoms, resulting in delayed diagnoses and limited therapeutic interventions. Only 15%-20% of PC patients present surgically resectable disease, but survival is significantly improved in these individuals (28–30). Therefore, early detection of surgically resectable lesions would enable timely interventions and potentially enhance clinical outcomes. PanIN, a neoplastic precursor lesion for PC, was proved to give rise to most PC (9). Unfortunately, it remains undetectable with current imaging modalities. An intriguing strategy to improve the sensitivity and specificity of imaging for detecting precursor lesions is the use of biomarkers. Although numerous potential blood biomarkers involving CTCs, ctDNA, exosomes, and serum proteins are currently under investigation, additional data are needed to better determine their clinical utility (13, 31–35). In this study, we further assessed the expression changes of TFF2 during the early stages of pancreatic carcinogenesis. By analyzing transcriptomic data from public datasets, we observed a significant upregulation of TFF2 expression in PanIN compared to normal pancreatic tissues, indicating that its dysregulation occurs early in pancreatic carcinogenesis. Our findings confirmed that TFF2 could serve as a promising serum biomarker for the early detection of PC, enabling the identification of high-risk individuals before the development of invasive cancer. However, the collection of clinical samples representing PanIN remains challenging, as these lesions are rarely detected without invasive procedures or the concurrent diagnosis of PC. This limitation prevented us from conducting validation studies using clinical serum samples from PanIN patients. Future studies should aim to overcome this challenge by utilizing longitudinal cohorts or experimental models that replicate early pancreatic carcinogenesis.

## Conclusion

In conclusion, our findings confirmed the robust diagnostic potential of CA199 in pancreatic cancer detection, but also revealed its limitations in distinguishing PC from periampullary adenocarcinoma. Moreover, our study identified a novel biomarker TFF2 that utilizes readily accessible blood samples to distinguish pancreatic cancer from periampullary adenocarcinoma. TFF2, an epithelial-derived secreted peptide aberrantly expressed during pancreatic tumorigenesis, promotes cell survival and malignant progression in the context of chronic inflammation and acinar-to-ductal metaplasia. These functions explain its elevated circulating levels in patients with PC and its specificity in distinguishing PC from periampullary adenocarcinoma. Importantly, it was also found that combining serum TFF2 with CA199 and CEA can enhance diagnostic accuracy, compensating for the shortcomings of CA199 and CEA. Certainly, rigorous, large-scale, and multicenter studies are necessary to further confirm its diagnostic utility across diverse patient populations. Together, our results position TFF2 as a biologically grounded and clinically actionable biomarker for the early detection and differential diagnosis of PC.

## Data Availability

The original contributions presented in the study are included in the article/**Supplementary Material**. Further inquiries can be directed to the corresponding author.
