# Supplementary material for: TFF2, a novel serum diagnostic biomarker for early pancreatic cancer
Source: Front Oncol. 2025 Sep 17;15:1633069. doi: 10.3389/fonc.2025.1633069 (PMC12484238; doi:10.3389/fonc.2025.1633069)
Supplement: Supplementary file 1 [file DataSheet1.docx]

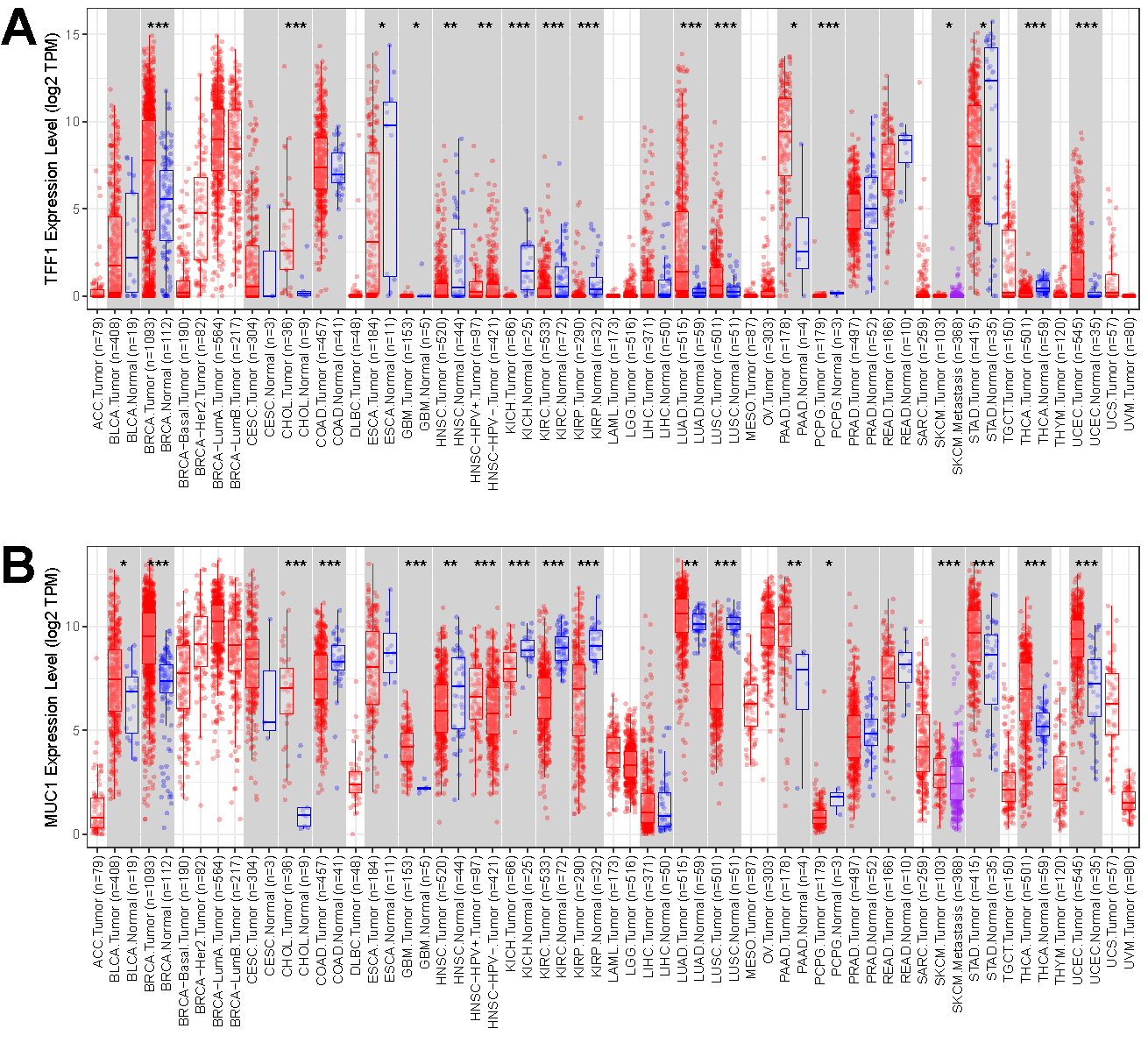


**Fig. S1** Pan-cancer analysis. (A, B) Gene expression levels of TFF1 and MUC1 in different cancers across TCGA.
