# Supplementary material for: TFF2, a novel serum diagnostic biomarker for early pancreatic cancer
Source: Front Oncol. 2025 Sep 17;15:1633069. doi: 10.3389/fonc.2025.1633069 (PMC12484238; doi:10.3389/fonc.2025.1633069)
Supplement: Supplementary file 3 [file Table2.docx]

**Supplementary Table 2** GEO datasets in the study

| **GEO datasets** | **Samples** |
| --- | --- |
| GSE43288 | 3 Normal pancreas |
|  | 13 Pancreatic intraepithelial neoplasia |
|  | 4 Pancreatic cancer |
| GSE15471 | 39 Pancreatic cancer |
|  | 39 Normal pancreas |
| GSE16515 | 36 Pancreatic cancer |
|  | 16 Normal pancreas |
| GSE32676 | 25 Pancreatic cancer |
|  | 7 Normal pancreas |
| GSE101462 | 6 Pancreatic cancer |
|  | 10 Pancreatitis |
| GSE43795 | 6 Pancreatic cancer |
|  | 14 Solid-pseudopapillary neoplasm |
| GSE143754 | 11 Pancreatic cancer |
| GSE76297 | 91 Cholangiocarcinoma |
|  | 62 Hepatocellular carcinoma |
| GSE26566 | 104 Cholangiocarcinoma |
|  | 6 Normal intrahepatic bile duct |
| GSE89377 | 40 Hepatocellular carcinoma |
|  | 13 Normal liver |
| GSE39409 | 8 Pancreatic cancer |
| GSE39409 | 2 Cholangiocarcinoma |
|  | 8 Duodenal adenocarcinoma |
|  | 14 ampullary adenocarcinoma |
| GSE155698 | 16 Pancreatic cancer |
|  | 3 Normal pancreas |
